# Supplementary material for: Key populations and healthcare providers perceptions, preferences and acceptability of HIV, Hepatitis B and C multiplex self-testing: A qualitative study
Source: PLoS One. 2026 May 12;21(5):e0346601. doi: 10.1371/journal.pone.0346601 (PMC13166938; doi:10.1371/journal.pone.0346601)
Supplement: S1 Table — (DOCX) [file pone.0346601.s001.docx]

**Supporting information**

S1 Table. Sociodemographic characteristics of participants in the optimization of IFU in Kyrgyzstan and Indonesia (Stage 2)

| Country | **Kyrgyzstan** | | | | | | | | **Indonesia** | | | | | | | |
| --- | --- | --- | --- | --- | --- | --- | --- | --- | --- | --- | --- | --- | --- | --- | --- | --- |
| **Type of participant** | **PWID** | | | | **HCPs** | | | | **PWID** | | | | **HCPs** | | | |
| **Type of ST evaluated** | **Dual ST** | | **Triple ST** | | **Dual ST** | | **Triple ST** | | **Dual ST** | | **Triple ST** | | **Dual ST** | | **Triple ST** | |
| **Number of participants** | ***N*=15** | | ***N*=15** | | ***N*=15** | | ***N*=15** | | ***N*=15** | | ***N*=15** | | ***N*=15** | | ***N*=13** | |
| **Age** (years) Mean [min, max] (SD) | 41.7 [29, 52] (7.27) | | 31.7 [22, 48] (7.29) | | 38.5 [28, 53] (8.77) | | 32.6 [26, 51] (7.11) | | 41.1 [29, 53] (6.73) | | 44.3 [39, 51] (4.3) | | 42.7 [30, 53] (6.47) | | 45.9 [35, 67] (9.03) | |
|  | *n* | **%** | *n* | **%** | *n* | **%** | *n* | **%** | *n* | **%** | *n* | **%** | *n* | **%** | *n* | **%** |
| **Gender** | | | | | | | | | | | | | | | | |
| Men | 10 | 67 | 11 | 73 | 10 | 67 | 10 | 67 | 15 | 100 | 14 | 93 | 5 | 33 | 5 | 38 |
| Women | 5 | 33 | 4 | 27 | 5 | 33 | 5 | 33 | 0 | 0 | 1 | 7 | 10 | 67 | 8 | 62 |
| **Education** |  |  |  |  |  |  |  |  |  |  |  |  |  |  |  |  |
| Elementary | 0 | 0 | 0 | 0 |  |  |  |  | 2 | 13 | 3 | 20 | 0 | 0 | 0 | 0 |
| Secondary | 4 | 26.7 | 2 | 13 | 2 | 13.3 | 0 | 0 | 11 | 0.73 | 6 | 40 | 1 | 7 | 5 | 39 |
| Vocational/  college | 8 | 53.3 | 10 | 67 | 13 | 86.7 | 9 | 60 | 1 | 7 | 3 | 20 | 5 | 33 | 4 | 31 |
| University or higher | 3 | 20 | 3 | 20 | 0 | 0 | 6 | 40 | 1 | 0.06 | 3 | 20 | 9 | 60 | 4 | 31 |
| **Employment** |  |  |  |  |  |  |  |  |  |  |  |  |  |  |  |  |
| Full time | 4 | 26.7 | 0 | 0 | 4 | 26.7 | 3 | 20 | 1 | 7 | 4 | 27 | 13 | 87 | 12 | 92 |
| Part time | 9 | 60 | 3 | 20 | 5 | 33.3 | 5 | 33 | 0 | 0 | 1 | 7 | 1 | 7 | 1 | 8 |
| Self employed | 2 | 13.3 | 5 | 33 | 5 | 33.3 | 5 | 33 | 10 | 67 | 6 | 40 | 0 | 0 | 0 | 0 |
| Homemaker | 0 | 0 | 7 | 47 | 0 | 0 | 2 | 13 | 0 | 0 | 1 | 7 | 0 | 0 | 0 | 0 |
| On leave/  unemployed | 0 | 0 | 0 | 0 | 1 | 6.7 | 0 | 0 | 4 | 27 | 3 | 20 | 1 | 7 | 0 | 0 |
| Residence | | | | | | | | | | | | | | | | |
| Urban | 12 | 80 | 13 | 87 | 12 | 80 | 11 | 73 | 10 | 67 | 8 | 53 | 10 | 67 | 8 | 62 |
| Rural | 3 | 20 | 2 | 13 | 3 | 20 | 4 | 27 | 5 | 0.33 | 7 | 47 | 5 | 33 | 5 | 39 |
