# Supplementary material for: Key populations and healthcare providers perceptions, preferences and acceptability of HIV, Hepatitis B and C multiplex self-testing: A qualitative study
Source: PLoS One. 2026 May 12;21(5):e0346601. doi: 10.1371/journal.pone.0346601 (PMC13166938; doi:10.1371/journal.pone.0346601)
Supplement: Attachment — Submitted filename: Reviewers comments-PONE-D-25-62011-PLOSE ONE.docx [file pone.0346601.s004.docx]

**Reviewers’ comments-** **PONE-D-25-62011**

This is an interesting qualitative study on ‘Key populations and healthcare providers perceptions, preferences and acceptability of HIV, Hepatitis B and C multiplex self-testing’. It is demonstrating initial acceptability of multiplex self-testing of HIV, Hepatitis B and C among people who inject drugs, health care providers, and stakeholders, which is aimed at generating evidence on instructions for use, as well as perceptions from potential end users that can impact implementation outcomes.

Generally, the manuscript is well written but below are a few suggestions to help improve its robustness.

1) My observation is that there are quite many abbreviations. For a phrase/word that appears very few times in the manuscript, I suggest you do not abbreviate. Also, abbreviations need to be expounded at first use, and some abbreviations need to be expanded for the reader to grasp meaning e.g. HCW (Pg 11, line 2)

2) Please indicate in your methods, what guided your sample selection? Was the 120 for cognitive interviews an additional sample or was this the same sample? This needs to be clearly reflected. How did the author(s) define ‘capacity to consent’?

3) How many FGDs were conducted and what was the size of the FGDs?

4) Pg 6 still in the methods, under ‘Instructions on use’, clearly explain what you mean by ‘No real IFUs were used’. Did participants have any experience of RDTs? If not, were participants asked to read the IFUs printed on the sites to ensure they read them? The authors should clearly indicate how this was done to ensure participants had information for the cognitive interviews.

5) The last statement under the ‘Data collection section’ needs revision to clearly indicate which people were part of the activity since the ‘No one …’ is confusing

6) Regarding the Analysis; who transcribed/verified the data? The authors need to clearly show how the data verification process was done to ensure its credibility and completeness. I suggest that the authors chronologically indicate how they identified the keys themes/codes.

7) In the Results section, paragraph 2 under ‘Perceptions on self-testing…’, line 1 of this paragraph indicates ‘inconvenient working hours’…the authors need to elaborate more on this by indicating the time that participants considered inconvenient and why.

8) Still under the Results, Table 1 looks congested and not easily readable. Secondly, it does not reflect the total number (overall) of participants to include participants in cognitive interviews. If these were the same participants for SSIs and FGDs, this needs to be clearly come out under ‘sampling and recruitment’ in the methods section.

9) I suggest that the authors systematically organise the results to clearly indicate the major themes and sub-themes and clearly organise the findings under the relevant themes.

10) Generally, the PWID voices do not seem to be strongly coming out of the data. We need to have more of their voices on the various aspects in the results.

11) The results for stage 2 need to have more depth, with supportive quotes following detailed analytical text of the findings instead of having stand-alone quotes.

12) In your Discussion, it would be good for the comparison of results from the two countries to appear in the results section rather than in the discussion. The discussion here should focus on the study findings in relation to other studies/ existing literature.
